# First order differential systems with a nonlinear boundary condition via the method of solution-regions

**DOI:** 10.1186/s13661-021-01495-9

**Published:** 2021-02-19

**Authors:** Marlène Frigon, Marcos Tella, F. Adrián F. Tojo

**Affiliations:** 1 Département de mathématiques et de statistique, Université de Montréal, Montreal, Canada; 2 Instituto de Matemáticas, Facultade de Matemáticas, Universidade de Santiago de Compostela, Santiago de Compostela, Spain

**Keywords:** 34L30, 34B15, Solution regions, Nonlinear boundary conditions, Existence results, Upper and lower solutions

## Abstract

In this article we extend the known theory of solution regions to encompass nonlinear boundary conditions. We both provide results for new boundary conditions and recover some known results for the linear case.

## Introduction

In this paper, we study systems of differential equations with nonlinear boundary conditions of the form:

1.1 $$ \begin{aligned} &u'(t) =f\bigl(t,u(t)\bigr) \quad\text{for a.e. } t\in I:=[0,T], \\ &L\bigl(u(0),u(T),u\bigr) = 0, \end{aligned} $$

where $f: [0,T] \times \mathbb{R}^{n} \to \mathbb{R}^{n}$ is a Carathéodory function and $L: \mathbb{R}^{2n}\times C(I,\mathbb{R}^{n}) \to \mathbb{R}^{n}$ is continuous.

To our knowledge, few results can be found in the literature in the case where $n > 1$ and *L* is nonlinear or does not depend only on the values at the boundary, $u(0)$ and $u(T)$. In [17] and [18], this problem was treated with linear integral boundary conditions. Results were obtained in the case where *f* satisfies a growth condition or a condition of contraction type.

The problem (1.1) in the case of a single differential equation ($n=1$) and *L* nonlinear of the form $L(x,y,u) = L(x,y)$ was studied by [1, 2, 4, 15]. Existence results were obtained with the method of upper and lower solutions and under monotonicity conditions imposed on *L*.

In a recent work of Frigon [7], the concept of solution-region was introduced for the first time in order to obtain results concerning the existence and multiplicity of solutions of the system of differential equations (1.1) in the particular case of the initial condition

$$ L\bigl(u(0),u(T),u\bigr) = u(0)-r=0, $$

or the periodic boundary condition

$$ L\bigl(u(0),u(T),u\bigr) = u(0)-u(T) = 0. $$

This method of solution-region was extended by Tojo in [20] to treat more general linear boundary conditions such as

$$ L\bigl(u(0),u(T),u\bigr)= \Gamma \bigl(u-u(0)\bigr)-r=0 \quad\text{or}\quad L \bigl(u(0),u(T),u\bigr)= \Gamma (u)-r=0, $$

where $r\in {\mathbb{R}^{n}}$ and $\Gamma: C([0,T],{\mathbb{R}^{n}})\to {\mathbb{R}^{n}}$ is a linear functional such that

$$ \Gamma (u_{1},\dots,u_{n}) = \bigl(\Gamma _{1}(u_{1}),\dots,\Gamma _{n}(u_{n}) \bigr) \quad\text{and}\quad \Gamma _{i}(1)\ne 0 \quad\text{for } i=1,\dots,n, $$

(with 1 understood as the constant function 1 on $[0,T]$).

This new method generalizes various means of obtaining existence and multiplicity of solutions of differential problems, such as the methods of upper and lower solutions [1, 2, 4, 9, 15, 16], strict upper and lower solutions [13, 19], and solution-tubes [3, 6, 8, 10]. Furthermore, the method is closely related to that of Gaines and Mawhin concerning what they called *bound sets* [11, 12]. The theory of bound sets was developed for the case where *f* is a continuous map and their existence results are obtained for bound sets not depending on *t*.

In this paper, we improve the works [7, 20] by studying systems of differential equations with nonlinear boundary conditions such as (1.1). The results obtained recover, in most cases, the results in [7, 20] for the case of linear boundary conditions. We also sharpen the definition and requirements for a set to be considered a solution-region (see Definition 3.1) for the case of nonlinear boundary conditions when compared to the linear one present in the literature (cf. [7, 20]). It is worthwhile to mention that no monotonicity or growth conditions will be imposed on *f* and *L*.

The paper is structured as follows. In Sect. 2 we deal with some preliminaries that we will use afterwards. Section 3 is concerned with the main results of this work and contains detailed applications that render already known results, thus showing the generality of our approach. Finally, in Sect. 4, we draw some conclusions regarding future work.

## Preliminaries

Throughout this paper, we consider ${\mathbb{R}}^{n}$ with the Euclidean norm $\|\cdot \|$, ${C}(\Omega, \mathbb{R}^{n})$, the space of continuous functions endowed with supremum norm $\|\cdot \|_{\infty }$, and $L^{1}(I,\mathbb{R})$ is the space of Lebesgue integrable functions endowed with the usual norm $\|\cdot \|_{1}$, where $I=[0,T]$ and Ω is some set. We consider also the Sobolev space $W^{1,1}(I,\mathbb{R}^{n})$ and, for $J \subset I$, the following set of locally absolutely continuous maps:

$$ W^{1,1}_{\operatorname{loc}}(J,\mathbb{R}) = \bigl\{ u: J \to \mathbb{R}: u \in W^{1,1}\bigl([t_{0},t_{1}],\mathbb{R}\bigr) \text{ for every } [t_{0},t_{1}] \subset J\bigr\} . $$

We recall the notion of locally Carathéodory functions introduced in [7].

### Definition 2.1

Let $D \subset I \times {\mathbb{R}}^{n}$. A map $f: D \to \mathbb{R}^{m}$ is a *Carathéodory function* if

(i) $f(t,\cdot )$ is continuous on $\{x: (t,x) \in D\}$ for almost every $t \in I$;
(ii) $f(\cdot,x)$ is measurable for all $x \in \{t: (t,x) \in D\}$;
(iii) for all $k > 0$, there exists $\psi _{k} \in L^{1}(I,\mathbb{R})$ such that $\|f(t,x)\| \leqslant \psi _{k}(t)$ for a.e. *t* and every *x* such that $\|x\| \leqslant k$ and $(t,x) \in D$.

A map $f: D \to \mathbb{R}^{m}$ is *locally Carathéodory* if $f|_{A}$ is a Carathéodory function for every compact set $A \subset D$.

It is well known that a completely continuous operator is associated to a Carathéodory map.

### Lemma 2.2

([5])

*Let* $g: I \times {\mathbb{R}}^{n} \to {\mathbb{R}}^{n}$ *be a Carathéodory function and* $N_{g}:{C}(I,{\mathbb{R}}^{n}) \to {C}(I,{\mathbb{R}}^{n})$ *the operator defined by*

2.1 $$ N_{g}(u) (t) = \int _{0}^{t} g\bigl(s,u(s)\bigr) \operatorname{d} s. $$

*Then*, $N_{g}$ *is continuous and completely continuous*.

The following comparison result will be useful to ensure that solutions are in a given set.

### Lemma 2.3

([7])

*Let* $w: [a,b] \to \mathbb{R}$ *be a continuous map and* $J = \{t \in [a,b]: w(t)>0\}$. *Assume that*

(i) $w \in W^{1,1}_{\operatorname{loc}}(J,\mathbb{R})$;
(ii) $w'(t) \leqslant 0$ *a*.*e*. $t \in J$; *and*
(iii) *one of the following conditions holds*:
  1. $w(a) \leqslant 0$;
  2. $w(a) \leqslant w(b)$.

*Then*, $w(t) \leqslant 0$ *for all* $t \in [a,b]$ *or there exists* $k>0$ *such that* $w(t)=k$ *for all* $t \in [a,b]$.

In order to present the problem of study, we recall the notion of an admissible region introduced in [7].

### Definition 2.4

([7])

We say that a set $R \subset I \times {\mathbb{R}}^{n}$ is an *admissible region* if there exist two continuous maps $h: I \times {\mathbb{R}}^{n} \to \mathbb{R}$ and $p = (p_{1},p_{2}): I \times {\mathbb{R}}^{n} \to I \times {\mathbb{R}}^{n}$ satisfying the following conditions:

1. $R = \{(t,x): h(t,x) \leqslant 0\}$ is bounded and, for every $t \in I$, $R_{t}=\{x\in {\mathbb{R}}^{n}: (t,x)\in R\}\ne \emptyset $;
2. the map *h* has partial derivatives at $(t,x)$ for almost every *t* and every *x* with $(t,x) \in R^{c}: = (I\times {\mathbb{R}}^{n} )\backslash R$, and $\frac{\partial h}{\partial t}$, $\nabla _{x}h$ are locally Carathéodory maps on $R^{c}$;
3. *p* is bounded and such that $p(t,x) =(t,x)$ for every $(t,x) \in R$ and
  $$ \bigl\langle \nabla _{x} h(t,x),p_{2}(t,x)-x \bigr\rangle < 0\quad \text{for a.e. $t$ and every $x$ with $(t,x) \in R^{c}$}. $$

We call $(h,p)$ an *admissible pair associated to* *R*.

In [20], a weaker notion of admissible region was considered where, in condition (H3), the inequality is taken to be non-strict.

### Definition 2.5

We say that a set $R \subset I \times {\mathbb{R}}^{n}$ is a *weak admissible region* if there exist two continuous maps $h: I \times {\mathbb{R}}^{n} \to \mathbb{R}$ and $p = (p_{1},p_{2}): I \times {\mathbb{R}}^{n} \to I \times {\mathbb{R}}^{n}$ satisfying (H1) and (H2) of Definition 2.4 and

(H3)’ *p* is bounded and such that $p(t,x) =(t,x)$ for every $(t,x) \in R$ and
  $$ \bigl\langle \nabla _{x} h(t,x),p_{2}(t,x)-x \bigr\rangle \leqslant 0, \quad\text{for a.e. $t$ and every $x$ with $(t,x) \in R^{c}$}. $$

We call $(h,p)$ a *weak admissible pair associated to* *R*.

## Solution-regions and nonlinear boundary conditions

We consider systems of first order differential equations of the form:

3.1 $$ u'(t) = f\bigl(t,u(t)\bigr)\quad \text{for a.e. } t\in [0,T], $$

subject to boundary conditions of the form $u \in {\mathcal{B}}$ where ${\mathcal{B}} $ denotes one of the following boundary conditions:

3.2 $$\begin{aligned} L\bigl(u(0),u(T),u\bigr) &=0; \end{aligned}$$

3.3 $$\begin{aligned} L\bigl(u(0),u(T),u\bigr) &= u(T)-u(0); \end{aligned}$$

where $f: [0,T]\times {\mathbb{R}}^{n}\to {\mathbb{R}}^{n}$ is a Carathéodory function and $L: \mathbb{R}^{2n}\times C([0,T],{\mathbb{R}}^{n}) \to {\mathbb{R}}^{n}$ is continuous but not necessarily linear. The boundary conditions (3.2) and (3.3) generalize those considered in [7].

We will be interested in giving conditions ensuring the existence of a solution of (3.1) in a suitable weak admissible region. To this end, we introduce the notion of solution-regions of (3.1).

### Definition 3.1

A set $R \subset I \times {\mathbb{R}}^{n}$ is called a *solution-region of* (3.1) if it is a weak admissible region with an associated weak admissible pair $(h,p)$ satisfying the following conditions:

(i) for almost every *t* and every *x* with $(t,x) \notin R$, one has
  3.4 $$ \frac{\partial h}{\partial t}(t,x) + \bigl\langle \nabla _{x} h(t,x),f\bigl(p(t,x)\bigr) \bigr\rangle \leqslant 0; $$
  and one of the following conditions:
(ii) If ${\mathcal{B}}$ denotes (3.2), for all $u \in W^{1,1}(I,{\mathbb{R}}^{n})$ such that $u(0)= p_{2} (0,u(0)-L(u(0),u(T),u) )$,
  - $h(0,u(0))\leqslant 0$;
  - $(0,u(0)-L(u(0),u(T),u) ) \in R$ if $(t,u(t)) \in R$ for every $t \in I$.
(ii)’ If ${\mathcal{B}}$ denotes (3.3),
  - $h(0,u(0)) \leqslant h(T,u(T))$ for every $u \in W^{1,1}(I,\mathbb{R}^{n})$ such that $(0,u(0)) \notin R$ and $u(T) - u(0) = tL((u(0),u(T),u))$ for some $t \in [0,1]$.
  - The inequality in (ii)’(a) is strict or there exists a set $S \subset I$ of positive measure such that one of the inequalities in (H3)’ or (3.4) is strict on *S*.

Now we show that the existence of a solution-region ensures the existence of a solution of (3.1).

### Theorem 3.2

*Let* $f: I \times {\mathbb{R}}^{n} \to {\mathbb{R}}^{n}$ *be a Carathéodory function*. *Assume that there exists a solution*-*region* *R* *of* (3.1). *Then*, *problem* (3.1), (3.2) *has a solution* $u \in W^{1,1}(I,{\mathbb{R}}^{n})$ *such that* $(t,u(t)) \in R$ *for every* $t \in I$.

### Proof

Let $(h,p)$ be a weak admissible pair associated to the solution-region *R*. For $\lambda \in [0,1]$, we consider the following family of problems: 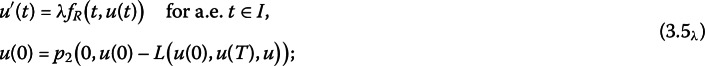 where $f_{R}: I \times \mathbb{R}^{n} \to \mathbb{R}^{n}$ is defined by

3.6 $$ f_{R}(t,x) = \textstyle\begin{cases} f(t,x) &\text{if $(t,x) \in R$,} \\ f(p(t,x)) + c(t)(p_{2}(t,x)-x) &\text{otherwise;} \end{cases} $$

with $c \in L^{1}(I,\mathbb{R})$ chosen such that

3.7 $$ c(t) > \bigl\Vert f\bigl(p(t,x)\bigr) \bigr\Vert \quad\text{for a.e. }t\in I\text{ and every }x \in \mathbb{R}^{n}. $$

Observe that such a function *c* exists since *f* is Carathéodory and *p* is a bounded map.

Let us consider the operators $\mathcal{I}_{0}: C(I,\mathbb{R}^{n}) \to \mathbb{R}^{n}$ and $\mathcal{I}: [0,1]\times C(I,\mathbb{R}^{n}) \to C(I,\mathbb{R}^{n})$ defined by

$$\mathcal{I}_{0}(u) = p_{2} \bigl(0,u(0)-L\bigl(u(0),u(T),u \bigr) \bigr) $$

and

$$\mathcal{I}(\lambda,u) = \mathcal{I}_{0}(u) + \lambda N_{f_{R}}(u), $$

where $N_{f_{R}}$ is defined in (2.1). Since *L* is continuous and *f* is Carathéodory, using (H3)’ of Definition 2.5, we deduce from Lemma 2.2 that $\mathcal{I}$ is continuous and completely continuous.

We claim that the fixed points of $\mathcal{I}$ are solutions of (3.5_*λ*_). Indeed, if $u = \mathcal{I}(\lambda,u)$,

$$ u(t) = \mathcal{I}_{0}(u) + \lambda \int _{0}^{t} f_{R}\bigl(s,u(s)\bigr)\, ds\quad \text{for every } t \in I. $$

In particular, one has, for $t=0$,

$$ u(0) = \mathcal{I}_{0}(u)= p_{2} \bigl(0,u(0)-L \bigl(u(0),u(T),u\bigr) \bigr), $$

and

$$ u'(t) = \lambda f_{R}\bigl(t,u(t)\bigr) \quad\text{for almost every $t \in I$}. $$

Thus, *u* is a solution of (3.5_*λ*_).

Fix $M > 0$ such that

3.8 $$ M > 1 + \Vert p_{2} \Vert _{\infty }. $$

We show that

3.9 $$ \Vert u \Vert _{\infty }< M \quad\text{for any solution $u$ of (3.5$_{\lambda }$)}. $$

Indeed, choose *m* such that

3.10 $$ M > m > 1 + \Vert p_{2} \Vert _{\infty }. $$

Assume that *u* is a solution of (3.5_*λ*_) for some $\lambda \in [0,1]$. If $\lambda =0$, $\|u(t)\|= \|\mathcal{I}_{0}(u)\| \leqslant \|p_{2}\|_{\infty }< m$. If $\lambda \in (0,1]$, then $\|u(0)\|= \|\mathcal{I}_{0}(u)\| \leqslant \|p_{2}\|_{\infty }< m$ and, from (3.6), (3.7), and (3.10), we deduce that, almost everywhere on $\{t \in I: \|u(t)\| > m\}$,

3.11 $$ \begin{aligned} \bigl\Vert u(t) \bigr\Vert ' &= \biggl\langle \frac{u(t)}{ \Vert u(t) \Vert },u'(t) \biggr\rangle \\ &= \biggl\langle \frac{u(t)}{ \Vert u(t) \Vert },\lambda f_{R}\bigl(t,u(t)\bigr) \biggr\rangle \\ &= \lambda \biggl\langle \frac{u(t)}{ \Vert u(t) \Vert },f\bigl(p\bigl(t,u(t)\bigr)\bigr) + c(t) \bigl(p_{2}\bigl(t,u(t)\bigr)-u(t)\bigr) \biggr\rangle \\ &\leqslant \lambda c(t) \bigl(1 + \bigl\Vert p_{2}\bigl(t,u(t)\bigr) \bigr\Vert - \bigl\Vert u(t) \bigr\Vert \bigr) \\ &< 0. \end{aligned} $$

Lemma 2.3 implies that $\|u(t)\| \leqslant m$ for every $t \in I$ since $\|u(0)\| \leqslant m$.

Let $\mathcal{U} = \{u \in C(I,\mathbb{R}^{n}): \|u\|_{\infty }< M\}$. It follows from (3.9) and the homotopy property of the fixed point index that

3.12 $$ \operatorname{index} \bigl(\mathcal{I}(\lambda,\cdot ), \mathcal{U} \bigr) = \operatorname{index} \bigl(\mathcal{I}(0,\cdot ), \mathcal{U} \bigr) \quad\text{for every } \lambda \in [0,1]. $$

Observe that

$$ \mathcal{I}(0,u) = \mathcal{I}_{0}(u) \in \mathbb{R}^{n} $$

and

$$ \mathcal{U}\cap \mathbb{R}^{n} = B_{\mathbb{R}^{n}}(0,M). $$

By the contraction property of the fixed point index (see [14, Chap. 4, Sect. 12, Theorem 6.2]), one has

3.13 $$ \operatorname{index} \bigl(\mathcal{I}(0,\cdot ),\mathcal{U} \bigr) = \operatorname{index} \bigl(\mathcal{I}_{0}(\cdot ),B_{\mathbb{R}^{n}}(0,M) \bigr). $$

Let us define $H_{0}: [0,1] \times \overline{B_{\mathbb{R}^{n}}(0,M)} \to \mathbb{R}^{n}$ by

$$ H_{0}(\lambda,x) = \lambda \mathcal{I}_{0}(x). $$

It is clear that $x \ne H_{0}(\lambda,x)$ for every $(\lambda,x) \in [0,1]\times \partial B_{\mathbb{R}^{n}}(0,M)$. The homotopy property of the fixed point index implies that

3.14 $$ \operatorname{index} \bigl(\mathcal{I}_{0}(\cdot ),B_{\mathbb{R}^{n}}(0,M) \bigr) = \operatorname{index} \bigl(H_{0}(0, \cdot ),B_{\mathbb{R}^{n}}(0,M) \bigr) = 1. $$

Combining (3.12), (3.13), and (3.14), one obtains

$$ \operatorname{index} \bigl(\mathcal{I}(\lambda,\cdot ),\mathcal{U} \bigr) = 1\quad \text{for every } \lambda \in [0,1]. $$

Therefore, for every $\lambda \in [0,1]$, $\mathcal{I}(\lambda,\cdot )$ has a fixed point, and hence (3.5_*λ*_) has a solution.

Now let *u* be a solution of (3.5_*λ*_) for $\lambda =1$. It follows from (3.6) and Definitions 2.5 and 3.1 that, almost everywhere on $\{t: h(t,u(t)) > 0\}$,

$$\begin{aligned} \frac{dh}{dt}\bigl(t,u(t)\bigr) &= \frac{\partial h}{\partial t}\bigl(t,u(t)\bigr) + \bigl\langle \nabla _{x} h\bigl(t,u(t)\bigr),u'(t) \bigr\rangle \\ &= \frac{\partial h}{\partial t}\bigl(t,u(t)\bigr) + \bigl\langle \nabla _{x} h \bigl(t,u(t)\bigr),f\bigl(p\bigl(t,u(t)\bigr)\bigr) + c(t) \bigl(p_{2} \bigl(t,u(t)\bigr)-u(t)\bigr) \bigr\rangle \\ &\leqslant 0. \end{aligned}$$

By Definition 3.1(ii)(a), $h(0,u(0))\leqslant 0$. Lemma 2.3 implies that $h(t,u(t))\leqslant 0$ for every $t\in I$, and hence, $(t,u(t)) \in R$ for every $t \in I$.

So, $u'(t)=f_{R}(t,x)=f(t,x)$ and equation (3.1) holds. Furthermore, by Definition 3.1(ii)(b), $(0,u(0)-L(u(0),u(T),u) )\in R$. Hence,

$$ u(0) = p_{2} \bigl(0,u(0)-L\bigl(u(0),u(T),u\bigr) \bigr) = u(0)-L \bigl(u(0),u(T),u\bigr). $$

Therefore, $L(u(0),u(T),u)=0$ and condition (3.2) holds. We conclude that *u* is a solution of (3.1), (3.2). □

We present an example of application of the previous theorem in which there are no monotonicity assumptions or growth conditions imposed on the right-hand side term of (3.1) or in (3.2).

### Example 3.3

We consider the following system of differential equations:

3.15 $$ \begin{aligned} &u_{1}'(t) = (t-3)u_{1}(t)u_{2}^{2}(t) + \frac{t^{2}}{4}, \\ &u_{2}'(t) = -u_{2}(t)e^{t+|u_{1}(t)|} + \frac{1-t^{2}}{4}, \quad\text{a.e. $t \in [0,1]$,} \\ &12u_{1}(0) + \int _{0}^{1}(6-5t)u_{1}(t)u_{2}(t) \operatorname{d} t = 0, \\ &3u_{2}(0)-u_{2}(1) = 0. \end{aligned} $$

Let $L: \mathbb{R}^{4}\times C([0,1],\mathbb{R}^{2}) \to \mathbb{R}^{2}$ be defined by

$$ L(x_{1},x_{2},y_{1},y_{2},u_{1},u_{2}) = \biggl(x_{1} + \frac{1}{12} \int _{0}^{1} (6-5t)u_{1}(t)u_{2}(t) \operatorname{d} t, \frac{3x_{2}-y_{2}}{4} \biggr). $$

We consider the closed and bounded set

$$ R = \biggl\{ (t,x_{1},x_{2}) \in [0,1]\times \mathbb{R}^{2}: \biggl( \biggl(1-\frac{t}{3} \biggr)x_{1} \biggr)^{2} + \biggl( \biggl(1- \frac{t}{2} \biggr)x_{2} \biggr)^{2} \leqslant 1 \biggr\} . $$

We define $h: [0,1] \times \mathbb{R}^{2} \to \mathbb{R}$ and $p: [0,1]\times \mathbb{R}^{2} \to [0,1]\times \mathbb{R}^{2}$ by

$$h(t,x_{1},x_{2}) = \biggl( \biggl( \biggl(1- \frac{t}{3} \biggr)x_{1} \biggr)^{2} + \biggl( \biggl(1-\frac{t}{2} \biggr)x_{2} \biggr)^{2} \biggr)^{\frac{1}{2}} -1, $$

and

$$p(t,x) = \textstyle\begin{cases} (t,x) &\text{if $(t,x) \in R$,} \\ (t,\frac{x}{h(t,x)+1} ) &\text{otherwise}. \end{cases} $$

It is easy to verify that *R* is an admissible region with the associated admissible pair $(h,p)$.

**Figure 1:**
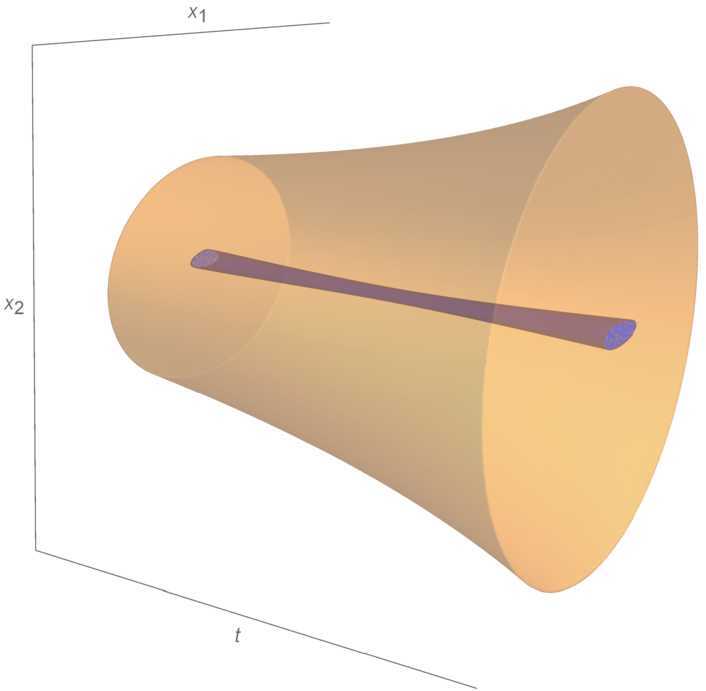
Region *R*. Inside, the smaller region where $\frac{\partial h}{\partial t}(t,x) + \langle \nabla _{x} h(t,x),f(p(t,x)) \rangle \geqslant 0$

To show that *R* is a solution-region of (3.15), we need to verify (3.4) (see Fig. 1). For $(t,x) \notin R$, one has

$$\begin{aligned} &\frac{\partial h}{\partial t}(t,x) + \bigl\langle \nabla _{x} h(t,x),f \bigl(p(t,x)\bigr) \bigr\rangle \\ &\quad= \frac{-1}{1+h(t,x)} \biggl( \frac{(3-t)}{9}x_{1}^{2} + \frac{(2-t)}{4}x_{2}^{2} \biggr) \\ &\qquad{} + \biggl\langle \biggl( \frac{ (1-\frac{t}{3} )^{2}x_{1}}{1+h(t,x)}, \frac{ (1-\frac{t}{2} )^{2}x_{2}}{1+h(t,x)} \biggr), \biggl(\frac{t^{2}}{4}+\frac{(t-3)x_{1}x_{2}^{2}}{(1+h(t,x))^{3}}, \frac{1-t^{2}}{4} - \frac{x_{2}e^{t+|x_{1}|}}{(1+h(t,x))} \biggr) \biggr\rangle \\ &\quad\leqslant \frac{-1}{1+h(t,x)} \biggl( \frac{(3-t)}{9}x_{1}^{2} + \frac{(2-t)}{4}x_{2}^{2} \biggr) + \biggl(1- \frac{t}{3} \biggr) \frac{t^{2}}{4} \biggl( \frac{ (1-\frac{t}{3} )x_{1}}{1+h(t,x)} \biggr) \\ & \qquad{}+ \biggl(1-\frac{t}{2} \biggr)\frac{1-t^{2}}{4} \biggl( \frac{( (1-\frac{t}{2} )x_{2}}{1+h(t,x)} \biggr). \end{aligned}$$

Observe that

$$ \biggl\vert \biggl(1-\frac{t}{3} \biggr)x_{1} \biggr\vert \leqslant 1 + h(t,x) \quad\text{and}\quad \biggl\vert \biggl(1-\frac{t}{2} \biggr)x_{2} \biggr\vert \leqslant 1 + h(t,x)\quad \forall (t,x)\notin R, $$

and

$$ \frac{3-t}{9} \geqslant \frac{1}{3} \biggl(1-\frac{t}{3} \biggr)^{2} \quad\text{and}\quad \frac{2-t}{4} \geqslant \frac{1}{3} \biggl(1- \frac{t}{2} \biggr)^{2} \quad\forall t \in [0,1]. $$

So,

$$\begin{aligned} &\frac{\partial h}{\partial t}(t,x) + \bigl\langle \nabla _{x} h(t,x),f \bigl(p(t,x)\bigr) \bigr\rangle \\ &\quad\leqslant \frac{-1}{3(1+h(t,x))} \biggl( \biggl(1-\frac{t}{3} \biggr)^{2}x_{1}^{2} + \biggl(1-\frac{t}{2} \biggr)^{2}x_{2}^{2} \biggr) + \biggl(1- \frac{t}{3} \biggr)\frac{t^{2}}{4}+ \biggl(1-\frac{t}{2} \biggr) \frac{(1-t^{2})}{4} \\ &\quad\leqslant \frac{-1}{3} \bigl(1+h(t,x) \bigr)^{2} + \frac{1}{4} - \frac{t}{8} + \frac{t^{3}}{24} \\ &\quad\leqslant \frac{-1}{3} + \frac{1}{4} \\ &\quad< 0. \end{aligned}$$

Observe that $h(0,p_{2}(0,x)) = h(p(0,x)) \leqslant 0$ for every $x \in \mathbb{R}^{2}$. In particular, if $u \in W^{1,1}([0,1],\mathbb{R}^{2})$ is such that $u(0) = p_{2} (0,u(0)-L(u(0),u(1),u) )$, then $h(0,u(0)) \leqslant 0$. In addition, if $(t,u(t)) \in R$ for every $t \in [0,1]$, then $|u_{1}(t)| \leqslant 3/(3-t)$, $|u_{2}(t)| \leqslant 2/(2-t)$,

$$ u(0)-L\bigl(u(0),u(1),u\bigr) = \biggl(\frac{1}{12} \int _{0}^{1}(6-5t)u_{1}(t)u_{2}(t) \operatorname{d} t,\frac{1}{4} \bigl(u_{2}(0)+u_{2}(1) \bigr) \biggr) $$

and

$$\begin{aligned} \bigl(1+h \bigl(0,u(0)-L\bigl(u(0),u(1),u\bigr) \bigr) \bigr)^{2} ={}& \biggl( \frac{1}{12} \int _{0}^{1}(6-5t)u_{1}(t)u_{2}(t) \operatorname{d} t \biggr)^{2} \\ &{} + \biggl(\frac{1}{4} \bigl(u_{2}(0)+u_{2}(1) \bigr) \biggr)^{2} \\ \leqslant{}& \biggl(\frac{1}{12} \int _{0}^{1} \frac{6(6-5t)}{(3-t)(2-t)} \operatorname{d} t \biggr)^{2} + \frac{1}{16}(1+2)^{2} \\ \leqslant{}& \biggl(\frac{1}{2} \biggr)^{2} + \frac{9}{16} \\ < {}&1. \end{aligned}$$

Hence, $(0,u(0)-L(u(0),u(1),u) ) \in R$. We have shown that condition (ii) of Definition 3.1 is satisfied. It follows from Theorem 3.2 that (3.15) has a solution $u \in W^{1,1}([0,1],\mathbb{R}^{2})$ such that $(t,u(t)) \in R$ for every $t \in [0,1]$.

As a corollary of the previous theorem, we obtain an existence result established in [7] for the initial value problem.

### Corollary 3.4

*Let* $R \subset I\times {\mathbb{R}}^{n}$ *be a weak admissible region*, $r \in {\mathbb{R}}^{n}$ *and* $f: I \times {\mathbb{R}}^{n} \to {\mathbb{R}}^{n}$ *be a Carathéodory function*. *Assume that there exists* $(h,p)$ *a weak admissible pair of* *R* *satisfying* (i) *of Definition* 3.1*and* $h(0,r) \leqslant 0$. *Then*, (3.1) *has a solution* $u \in W^{1,1}(I,{\mathbb{R}^{n}})$ *such that* $(t,u(t)) \in R$ *for every* $t \in I$, *and* $u(0)=r$.

### Proof

Observe that the initial value problem

$$\begin{aligned} u'(t)= {}& f\bigl(t,u(t)\bigr)\quad\text{for a.e. } t\in I, \\ u(0)={}&r \end{aligned}$$

is the problem (3.1), (3.2) with $L(x,y,u)=x-r$. The conclusion will follow from Theorem 3.2 if *R* is a solution region of (3.1), (3.2). We only need to verify condition (ii) of Definition 3.1. Observe that

$$ \bigl(0,x-L(x,y,u)\bigr) = \bigl(0,x-(x-r)\bigr) = (0,r). $$

By assumption, $h(0,r) \leqslant 0$. So, $(0,r) \in R$ and Definition 3.1(ii)(b) is satisfied. Moreover, $p(0,r)=(0,r)$. Therefore, the condition $u(0)= p_{2} (0,u(0)-L(u(0),u(T),u) )$ in Definition 3.1(ii)(a) turns into $u(0)=r$. Hence, $h(0,u(0))\leqslant 0$ and the result holds. □

Theorem 3.2 has also, as a corollary, an existence result for a first order differential equation with a nonlinear boundary condition and under a condition of existence of lower and upper solutions.

### Corollary 3.5

*Let* $f: I \times \mathbb{R} \to \mathbb{R}$ *be a Carathéodory function*, $L: \mathbb{R}^{2}\times {C}(I,\mathbb{R}) \to \mathbb{R}$ *be continuous and* $\alpha, \beta \in W^{1,1}(I,\mathbb{R})$ *such that*

(i) $\alpha (t) \leqslant \beta (t)$ *for every* $t \in I$;
(ii) $f(t,\beta (t)) \leqslant \beta '(t)$ *for almost every* $t \in I$; *and* $L(\beta (0),\beta (T),\beta ) \geqslant 0$;
(iii) $f(t,\alpha (t)) \geqslant \alpha '(t)$ *for almost every* $t \in I$; *and* $L(\alpha (0),\alpha (T),\alpha ) \leqslant 0$;
(iv) $L(\alpha (0),u(t),u) \leqslant L(\alpha (0),\alpha (T),\alpha )$ *for every* $u \in {C}(I,\mathbb{R})$ *such that* $u(0)= \alpha (0)$ *and* $\alpha (t) \leqslant u(t) \leqslant \beta (t)$ *for every* $t \in I$;
(v) $L(\beta (0),u(t),u) \geqslant L(\beta (0),\beta (T),\beta )$ *for every* $u \in {C}(I,\mathbb{R})$ *such that* $u(0)= \beta (0)$ *and* $\alpha (t) \leqslant u(t) \leqslant \beta (t)$ *for every* $t \in I$.

*Then*, (3.1), (3.2) *has a solution* $u \in W^{1,1}(I,{\mathbb{R}})$ *such that* $(t,u(t)) \in R$ *for every* $t \in I$, *with*

$$ R= \bigl\{ (t,x) \in I \times \mathbb{R}: \alpha (t) \leqslant x \leqslant \beta (t)\bigr\} . $$

### Proof

As pointed out in [7] (see Examples 3.2 and 4.2), $(h,p)$ where

$$h(t,x) = \biggl\vert x-\frac{\beta (t)+\alpha (t)}{2} \biggr\vert - \frac{\beta (t)-\alpha (t)}{2}= \textstyle\begin{cases} x-\beta (t) & \text{if } x\geqslant \frac{\alpha (t)+\beta (t)}{2}, \\ \alpha (t)-x & \text{if } x\leqslant \frac{\alpha (t)+\beta (t)}{2}, \end{cases} $$

and

$$p(t,x) = \textstyle\begin{cases} (t,x) &\text{if $\alpha (t) \leqslant x \leqslant \beta (t)$,} \\ (t,\beta (t)) &\text{if $x > \beta (t)$,} \\ (t,\alpha (t)) &\text{if $x < \alpha (t)$,} \end{cases} $$

is an admissible pair of *R* satisfying Definition 3.1(i). Indeed, $h^{-1}((-\infty,0])=R$ and, by definition of *α* and *β*, (H2) holds as well. Finally, for a.e. *t* and every *x* with $(t,x) \in R^{c}$, we study two different cases:

- If $x<\alpha (t)$, by (iii),
  $$\begin{aligned} & \bigl\langle \nabla _{x} h(t,x),p_{2}(t,x)-x \bigr\rangle =-\bigl( \alpha (t)-x\bigr)< 0 \quad\text{and} \\ &\frac{\partial h}{\partial t}(t,x) + \bigl\langle \nabla _{x} h(t,x),f \bigl(p(t,x)\bigr) \bigr\rangle =\alpha '(t)-f\bigl(t,\alpha (t) \bigr)\leqslant 0; \end{aligned}$$
- If $x>\beta (t)$,
  $$\begin{aligned} & \bigl\langle \nabla _{x} h(t,x),p_{2}(t,x)-x \bigr\rangle =x- \beta (t)< 0 \quad\text{and} \\ &\frac{\partial h}{\partial t}(t,x) + \bigl\langle \nabla _{x} h(t,x),f \bigl(p(t,x)\bigr) \bigr\rangle =-\beta '(t)+f\bigl(t,\alpha (t) \bigr)\leqslant 0. \end{aligned}$$

Hence, $(h,p)$ is an admissible pair and Definition 3.1(i) holds. Finally, observe that $p_{2}(0,x) \in [\alpha (0),\beta (0)]$ for every $x \in \mathbb{R}$. Therefore,

$$\begin{aligned} &h \bigl(0,u(0) \bigr) \leqslant 0 \\ &\quad\text{for every } u \in W^{1,1}(I, \mathbb{R})\text{ such that }u(0) = p_{2} \bigl(0,u(0)-L\bigl(u(0),u(T),u \bigr) \bigr). \end{aligned}$$

Hence, Definition 3.1(ii)(a) is satisfied.

Let $u \in W^{1,1}(I,\mathbb{R})$ be such that

$$ \alpha (t) \leqslant u(t)\leqslant \beta (t)\quad \forall t \in I \quad\text{and}\quad u(0) = p_{2} \bigl(0,u(0)-L\bigl(u(0),u(T),u\bigr) \bigr). $$

Assume that $(0,u(0)-L(u(0),u(T),u) ) \notin R$. Without loss of generality,

$$ u(0) -L\bigl(u(0),u(T),u\bigr) < \alpha (0). $$

Then,

$$ u(0) = p_{2} \bigl(0,u(0)-L\bigl(u(0),u(T),u\bigr) \bigr) = \alpha (0), $$

and, by (iii) and (iv),

$$ 0 < L\bigl(u(0),u(T),u\bigr) = L\bigl(\alpha (0),u(T),u\bigr) \leqslant L\bigl( \alpha (0), \alpha (T),\alpha \bigr) \leqslant 0, $$

which is a contradiction. Similarly, we get a contradiction if we assume that $u(0) -L(u(0),u(T),u) > \beta (0)$. So, condition (ii)(b) of Definition 3.1 is satisfied.

Therefore, *R* is a solution-region of (3.1), (3.2) and the conclusion follows from Theorem 3.2. □

The next corollary is similar (although not comparable) to [20, Theorem 4.9].

### Corollary 3.6

*Let* $R \subset I\times {\mathbb{R}}^{n}$ *be a weak admissible region*, $\Gamma:{C}([a,b],{\mathbb{R}})\to {\mathbb{R}^{n}}$ *continuous and* $f: I \times {\mathbb{R}}^{n} \to {\mathbb{R}}^{n}$ *be a Carathéodory function*. *Assume that there exists a weak admissible pair* $(h,p)$ *of* *R* *satisfying* (i) *of Definition* 3.1*and*

(ii) $h(0,p_{2}(0,x)) \leqslant 0$ *for all* $x \in {\mathbb{R}}^{n}$;
(iii) $(0,u(0) + \Gamma (u) ) \in R$ *for every* $u \in W^{1,1}(I,\mathbb{R}^{n})$ *such that* $(t,u(t)) \in R$ *for every* $t \in I$, *and* $u(0)=p_{2} (0,u(0) + \Gamma (u) )$.

*Then*, (3.1) *has a solution* $u \in W^{1,1}(I,{\mathbb{R}^{n}})$ *such that* $(t,u(t)) \in R$ *for every* $t \in I$ *and* $\Gamma (u) = 0$.

### Proof

Let us consider the problem (3.1), (3.2) with $L(x,y,u)=L(x,u)=-\Gamma (u)$.

To show that *R* is a solution-region of (3.1), (3.2), we only need to verify condition (ii) of Definition 3.1. Let $u \in W^{1,1}(I,{\mathbb{R}}^{n})$ be such that $u(0)=p_{2} (0,u(0)-L(u(0),u) )$. Then

$$ u(0)=p_{2} \bigl(0,u(0)-L\bigl(u(0),u\bigr) \bigr)=p_{2} \bigl(0,u(0)+\Gamma (u)\bigr). $$

Therefore, by (ii),

$$ h \bigl(0,u(0) \bigr)=h \bigl(0,p_{2}\bigl(0,u(0)+\Gamma (u)\bigr) \bigr) \leqslant 0, $$

and, by (iii),

$$ \bigl(0,u(0)-L\bigl(u(0),u\bigr) \bigr) = \bigl(0,u(0)+\Gamma (u) \bigr) \in R $$

if, in addition, $(t,u(t)) \in R$ for every $t \in I$. So, Definition 3.1(ii) is satisfied. Thus, *R* is a solution-region of (3.1), (3.2). Theorem 3.2 ensures the existence of $u \in W^{1,1}(I,\mathbb{R}^{n})$ a solution of (3.1) such that $(t,u(t)) \in R$ for every $t \in I$ and $\Gamma (u)=0$. □

Here is another corollary of Theorem 3.2 in which a solution of (3.1), (3.2) with the periodic boundary condition is obtained.

### Corollary 3.7

*Let* $f: I \times \mathbb{R}^{n} \to \mathbb{R}^{n}$ *be a Carathéodory function and* $R \subset I\times \mathbb{R}^{n}$ *a weak admissible region with an associated weak admissible pair* $(h,p)$ *satisfying* (i) *of Definition *3.1*and*

(ii) $h(0,p_{2}(0,x)) \leqslant 0$ *for all* $x \in \mathbb{R}^{n}$;
(iii) $h(0,x) \leqslant h(T,x)$ *for every* *x* *such that* $(0,x) \notin R$.

*Then*, (3.1) *has a solution* $u \in W^{1,1}(I,{\mathbb{R}^{n}})$ *such that* $(t,u(t)) \in R$ *for every* $t \in I$ *and* $u(0)=u(T)$.

### Proof

We consider the problem (3.1), (3.2) with $L(x,y,u)=x-y$. To show that *R* is a solution-region, one needs to verify condition (ii) of Definition 3.1. Observe that

$$ x-L(x,y,u)= x-(x-y) = y. $$

Let $u \in W^{1,1}(I,\mathbb{R}^{n})$ be such that $u(0)= p_{2} (0,u(0)-L(u(0),u(T),u) )= p_{2}(0,u(T))$. By (ii), we have that

$$ h\bigl(0,u(0)\bigr)= h\bigl(0,p_{2}\bigl(0,u(T)\bigr)\bigr)\leqslant 0. $$

In addition, if $(t,u(t)) \in R$ for every $t \in I$ and if $(0,u(0)-L(u(0),u(T),u) ) =(0,u(T)) \notin R$, then, by (iii),

$$ 0 < h\bigl(0,u(T)\bigr) \leqslant h\bigl(T,u(T)\bigr). $$

This contradicts the fact that $(T,u(T)) \in R$. Thus, *R* is a solution region of (3.1), (3.2) with $L(x,y,u) = x-y$. The conclusion follows from Theorem 3.2. □

### Remark 3.8

If $p(0,\cdot )$ is a projection onto $\{(0,x) \in R\}$, then condition (ii) in Corollaries 3.6 and 3.7 is satisfied.

Observe that, for $L(x,y,u) = x-y$, the last corollary does not recover the results in [7] for the periodic problem because of condition (ii). The next theorem will show that (ii) of Corollary 3.7 is not necessary if *R* is an admissible region or if the inequality in Definition 3.1(i) is strict (see Definition 3.1(ii)’(b)).

### Theorem 3.9

*Let* $f: I \times \mathbb{R}^{n} \to \mathbb{R}^{n}$ *be a Carathéodory function*. *Assume that there exists* *R*, *a solution*-*region of* (3.1) *with the boundary condition* (3.3). *Then*, *the problem* (3.1), (3.3) *has a solution* $u \in W^{1,1}(I,\mathbb{R}^{n})$ *such that* $(t,u(t)) \in R$ *for every* $t \in I$.

### Proof

Let $(h,p)$ be a weak admissible pair associated to the solution-region *R*. For $\lambda \in [0,1]$, we consider the following family of problems: 
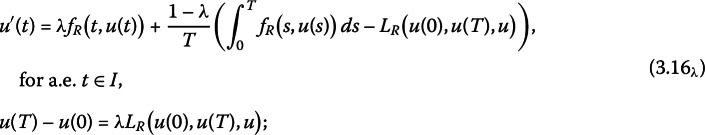
 where $f_{R}: I \times \mathbb{R}^{n} \to \mathbb{R}^{n}$ is defined in (3.6) and $L_{R}: \mathbb{R}^{2n} \times {C}(I,\mathbb{R}^{n}) \to \mathbb{R}^{n}$ is given by

3.17 $$ L_{R}(x,y,u) = \textstyle\begin{cases} L(x,y,u) &\text{if $ \Vert x \Vert \leqslant m_{0}: =\max \{ \Vert z \Vert : (0,z) \in R\}$,} \\ (1-t)L(x,y,u) &\text{if $ \Vert x \Vert = m_{0}+t$ for $t \in (0,1)$,} \\ 0 &\text{otherwise.} \end{cases} $$

Observe that, integrating the equation in (3.16_*λ*_) between 0 and *T*, we have that

$$\begin{aligned} u(T)-u(0)={} & \lambda \int _{0}^{T}f_{R}\bigl(s,u(s)\bigr)\, ds+ (1-\lambda ) \biggl( \int _{0}^{T} f_{R}\bigl(s,u(s)\bigr)\, ds - L_{R}\bigl(u(0),u(T),u\bigr) \biggr) \\ ={}& \int _{0}^{T}f_{R}\bigl(s,u(s)\bigr)\, ds- (1-\lambda ) L_{R}\bigl(u(0),u(T),u\bigr), \end{aligned}$$

which, combined with the boundary conditions in (3.16_*λ*_), yields

3.18 $$ N_{f_{R}}(u) (T) = L_{R}\bigl(u(0),u(T),u \bigr). $$

Let us consider the operator $\mathcal{P}: [0,1]\times {C}(I,\mathbb{R}^{n}) \to {C}(I,\mathbb{R}^{n})$ defined by

$$ \mathcal{P}(\lambda,u) (t) = u(0) + \lambda N_{f_{R}}(u) (t) - \frac{(1+\lambda t)}{T} \bigl( N_{f_{R}}(u) (T) - L_{R} \bigl(u(0),u(T),u\bigr) \bigr), $$

where $N_{f_{R}}$ is defined in (2.1). As in the proof of the previous theorem, we deduce that $\mathcal{P}$ is continuous and completely continuous.

We claim that the fixed points of $\mathcal{P}$ are solutions of (3.16_*λ*_). Indeed, if $u = \mathcal{P}(\lambda,u)$, then, for every $t \in I$,

3.19 $$ u(t) = u(0) - \frac{(1+\lambda t)}{T} \bigl( N_{f_{R}}(u) (T) - L_{R}\bigl(u(0),u(T),u\bigr) \bigr) +\lambda \int _{0}^{t} f_{R}\bigl(s,u(s)\bigr) \operatorname{d} s. $$

In particular, for $t=0$ and for $t=T$, we have

$$\begin{aligned} &u(0) = u(0) - \frac{1}{T} N_{f_{R}}(u) (T) +\frac{1}{T}L_{R} \bigl(u(0),u(T),u\bigr), \\ &u(T) = u(0) - \frac{1}{T} N_{f_{R}}(u) (T)+ \biggl( \frac{1}{T}+ \lambda \biggr)L_{R}\bigl(u(0),u(T),u\bigr). \end{aligned}$$

Thus,

3.20 $$ u(T)-u(0) = \lambda L_{R}\bigl(u(0),u(T),u\bigr), $$

and (3.18) holds. From (3.18) and (3.19), we deduce that, for almost every $t \in I$,

$$\begin{aligned} u'(t) &= \lambda f_{R}\bigl(t,u(t)\bigr) + \frac{1-\lambda }{T} \bigl( N_{f_{R}}(u) (T) - L_{R} \bigl(u(0),u(T),u\bigr) \bigr). \end{aligned}$$

Thus, *u* is a solution of (3.16_*λ*_).

Let *M* and *m* be as in (3.8) and (3.10), respectively. Assume that *u* is a solution of (3.16_*λ*_) for some $\lambda \in [0,1]$. If $\lambda =0$ then

$$\begin{aligned} &u'(t) = \frac{1}{T} \biggl( \int _{0}^{T} f_{R}\bigl(s,u(s)\bigr) \, ds- L_{R}\bigl(u(0),u(T),u\bigr) \biggr) \quad\text{for a.e. } t \in I, \\ &u(T)-u(0)=0, \end{aligned}$$

that is, $u'$ is constant and $u(T)=u(0)$, so *u* is constant, say $u \equiv k$, and

$$ \int _{0}^{T} f_{R}\bigl(s,u(s)\bigr) \, ds= L_{R}\bigl(u(0),u(T),u\bigr). $$

If $\|k\| > m >1+\|p_{2}\|_{\infty }\geqslant m_{0}+1$, by (3.17),

$$ 0 = L_{R}(k,k,k) = \int _{0}^{T} f_{R}(t,k)\,dt = \int _{0}^{T} \bigl[f\bigl(p(t,k)\bigr) + c(t) \bigl( p_{2}(t,k)-k \bigr)\bigr]\,dt, $$

that is,

$$ k \int _{0}^{T} c(t)\,dt= \int _{0}^{T} \bigl(f\bigl(p(t,k)\bigr)+ c(t)p_{2}(t,k) \bigr)\,dt. $$

Taking norms on both sides of this equality and combining it with (3.7) and (3.10), we obtain

$$ \Vert k \Vert \Vert c \Vert _{L^{1}} \leqslant \int _{0}^{T} c(t) \bigl(1+ \bigl\Vert p_{2}(t,k) \bigr\Vert \bigr) \,dt\leqslant \int _{0}^{T} c(t) \bigl(1+ \Vert p_{2} \Vert _{\infty }\bigr)\,dt < m \Vert c \Vert _{L^{1}}, $$

which is a contradiction, so $\|u(t)\|=\|k\|\leqslant m< M$ for every $t\in I$.

On the other hand, if $\lambda \in (0,1]$, combining (3.6), (3.7), (3.10), and (3.18) allows us to deduce that, as in (3.11),

$$ \bigl\Vert u(t) \bigr\Vert ' < 0 \quad\text{a.e. on $\bigl\{ t \in I: \bigl\Vert u(t) \bigr\Vert > m\bigr\} $.} $$

By (3.17) and (3.20), we have that either $\|u(0)\| \leqslant m$ or $m<\|u(0)\| = \|u(T)\|$. Taking this into account, it follows from Lemma 2.3 that $\|u(t)\| \leqslant m$ for every $t \in I$ since $\|u\|$ cannot be constant and greater than *m*. Hence,

3.21 $$ \Vert u \Vert _{\infty }< M\quad \text{for any solution $u$ of (3.16$_{\lambda }$)}. $$

Let $\mathcal{U} = \{u \in {C}(I,\mathbb{R}^{n}): \|u\|_{\infty }< M\}$. It follows from (3.21) and the homotopy property of the fixed point index that

3.22 $$ \operatorname{index} \bigl( \mathcal{P}(\lambda,\cdot ), \mathcal{U} \bigr) = \operatorname{index} \bigl( \mathcal{P}(0,\cdot ), \mathcal{U} \bigr) \quad\text{for every } \lambda \in [0,1]. $$

Observe that

$$ \mathcal{P}(0,u) = u(0) - \frac{1}{T} \bigl( N_{f_{R}}(u) (T) - L_{R}\bigl(u(0),u(T),u\bigr) \bigr) \in \mathbb{R}^{n} $$

and

$$ \mathcal{U}\cap \mathbb{R}^{n} = B_{\mathbb{R}^{n}}(0,M). $$

By the contraction property of the fixed point index, one has

3.23 $$ \operatorname{index} \bigl( \mathcal{P}(0,\cdot ),\mathcal{U} \bigr) = \operatorname{index} \bigl( \mathcal{P}(0,\cdot ),B_{\mathbb{R}^{n}}(0,M) \bigr). $$

Let us define $P_{0}: [0,1] \times \overline{B_{\mathbb{R}^{n}}(0,M)} \to \mathbb{R}^{n}$ by

$$ P_{0}(\lambda,x) = \lambda \mathcal{P}(0,x) + 2(1-\lambda )x. $$

We claim that $x \ne P_{0}(\lambda,x)$ for every $(\lambda,x) \in [0,1]\times \partial B_{\mathbb{R}^{n}}(0,M)$. Indeed, for $\lambda =0$ this fact is evident and, if $\lambda \in (0,1]$, assume that there exists $x \in \mathbb{R}^{n}$ such that $\|x\|=M$ and $x=P_{0}(\lambda,x)$. By (3.6), (3.7), (3.8), and (3.17), it satisfies

$$\begin{aligned} x=P_{0}(\lambda,x) &= 2(1-\lambda )x + \lambda \biggl(x - \frac{1}{T} \int _{0}^{T} f_{R}(s,x) \operatorname{d} s \biggr) \\ &= 2(1-\lambda )x + \lambda \biggl(x - \frac{1}{T} \int _{0}^{T} \bigl[f\bigl(p(s,x)\bigr) +c(s) \bigl(p_{2}(s,x)-x\bigr)\bigr] \operatorname{d} s \biggr) \\ &= \biggl(2-\lambda +\frac{\lambda \Vert c \Vert _{L^{1}}}{T} \biggr)x - \frac{\lambda }{T} \int _{0}^{T}\bigl[ f\bigl(p(s,x)\bigr) +c(s)p_{2}(s,x)\bigr] \operatorname{d} s. \end{aligned}$$

So, taking norms on both sides,

$$\begin{aligned} M \biggl(1-\lambda +\frac{\lambda \Vert c \Vert _{L^{1}}}{T} \biggr) &= \biggl(1- \lambda + \frac{\lambda \Vert c \Vert _{L^{1}}}{T} \biggr) \Vert x \Vert = \biggl\Vert \frac{\lambda }{T} \int _{0}^{T} \bigl[f\bigl(p(s,x)\bigr) +c(s)p_{2}(s,x)\bigr] \operatorname{d} s \biggr\Vert \\ &< \frac{\lambda }{T} \int _{0}^{T}\bigl[ c(s) +c(s) (M-1)\bigr] \operatorname{d} s = \frac{M\lambda \Vert c \Vert _{L^{1}}}{T}, \end{aligned}$$

which is a contradiction.

The homotopy property of the fixed point index implies that

3.24 $$ \begin{aligned}\operatorname{index} \bigl( P_{0}(1,\cdot ),B_{\mathbb{R}^{n}}(0,M) \bigr) &= \operatorname{index} \bigl( P_{0}(0, \cdot ),B_{\mathbb{R}^{n}}(0,M) \bigr)\\ &= \operatorname{index} \bigl( 2 \text{Id},B_{\mathbb{R}^{n}}(0,M) \bigr) = (-1)^{n}. \end{aligned}$$

Combining (3.22), (3.23), and (3.24), we obtain

$$ \operatorname{index} \bigl( \mathcal{P}(\lambda,\cdot ),\mathcal{U} \bigr) = (-1)^{n}\quad \text{for every } \lambda \in [0,1]. $$

Therefore, for every $\lambda \in [0,1]$, $\mathcal{P}(\lambda,\cdot )$ has a fixed point, and hence (3.16_*λ*_) has a solution.

Let *u* be a solution of (3.16_*λ*_) for $\lambda =1$. It follows from (3.6) and Definitions 2.5 and 3.1 that, almost everywhere on $\{t: h(t,u(t)) > 0\}$, one has

3.25 $$ \begin{aligned}& \frac{dh}{dt}\bigl(t,u(t)\bigr) \\ &\quad= \frac{\partial h}{\partial t}\bigl(t,u(t)\bigr) + \bigl\langle \nabla _{x} h \bigl(t,u(t)\bigr),u'(t) \bigr\rangle \\ &\quad= \frac{\partial h}{\partial t}\bigl(t,u(t)\bigr) + \bigl\langle \nabla _{x} h \bigl(t,u(t)\bigr),f\bigl(p\bigl(t,u(t)\bigr)\bigr) + c(t) \bigl(p_{2} \bigl(t,u(t)\bigr)-u(t)\bigr) \bigr\rangle \leqslant 0. \end{aligned} $$

Since $u(T)-u(0)=L_{R}(u(0),u(T),u)$, we have that

$$ u(T) = u(0) + sL\bigl(u(0),u(T),u\bigr)\quad \text{for some $s \in [0,1]$.} $$

If $h(0,u(0)) > 0$, we deduce from Definition 3.1(ii)’(a) that $h(0,u(0)) \leqslant h(T,u(T))$. Moreover, by Definition 3.1(ii)’(b), this last inequality is strict or the inequality (3.25) is strict on a subset of positive measure. This prevents the existence of $k>0$ such that $h(t,u(t)) = k$ for every $t\in I$. Thus, Lemma 2.3 implies that $(t,u(t)) \in R$ for every $t \in I$. Therefore, *u* is a solution of (3.1), (3.3) since $p=\text{Id}$ on *R* and $L_{R}(u(0),u(T),u) = L(u(0),u(T),u)$. □

## Conclusions

In this work, we have extended the known theory of solution regions to encompass nonlinear boundary conditions and, at the same time, we have recovered some known results for the linear case from our generalization. This was achieved by refining the definition of solution-region for the case of a weak admissible region and nonlinear boundary conditions. Nonetheless, some of the requirements in the definition (see Definition 3.1(ii)’(b)) arise from the fact that in the proof of Theorem 3.9 (see last paragraph) the properties of a weak admissible region are not enough to conclude, something which was indeed the case when solutions regions where defined from admissible regions (cf. [7, Theorem 5.1]).

This raises the question as to whether or why consider weak admissible regions altogether. This laxer definition appeared in [20] in order to prove the following result.

### Theorem 4.1

([20])

*Let* $R \subset I \times {\mathbb{R}}^{n}$ *be a compact set such that the projection of* *R* *onto* *I* *is surjective*. *Then*, *R* *is a weak admissible region*.

This way, weak admissible regions are characterized in a simple topological way, which simplifies the application of the theory. In [20], it was already conjectured that, in fact, all weak admissible regions are admissible regions for an adequate admissible pair. Unfortunately, to the best of our knowledge, there is no proof of that fact as of today. Such a proof would simplify the theory since we could profit from a simple characterization and, at the same time, a simpler definition of solution regions, so it is a result to look forward to.

## Data Availability

Not applicable.
